# A Unique Case of Compressive Optic Neuropathy From an Internal Carotid Artery Aneurysm

**DOI:** 10.1155/crop/6458555

**Published:** 2025-10-25

**Authors:** Daniel S. Boyes, Kiren Bashir, Jonathan M. Skarie, Ryan E. Chenevey

**Affiliations:** ^1^Wright State University Boonshoft School of Medicine, Fairborn, Ohio, USA; ^2^Ohio Eye Associates, Mansfield, Ohio, USA; ^3^Department of Population and Quantitative Health Sciences, School of Medicine, Case Western Reserve University, Cleveland, Ohio, USA; ^4^Wooster Eye Center, Wooster, Ohio, USA

**Keywords:** compressive optic neuropathy, internal carotid artery aneurysm, junctional scotoma, relative afferent pupillary defect

## Abstract

Compressive optic neuropathy (CON) is a condition characterized by optic nerve damage caused by abnormal mechanical pressure. Here, we report a rare case of bilateral CON due to a large, right supraclinoid internal carotid artery (ICA) aneurysm. A 66-year-old female presented to the clinic with complaints of prior, intermittent headaches and decreased visual acuity. Notably, no relative afferent pupillary defect (RAPD) was present on exam. Visual field testing revealed an inferior altitudinal defect oculus dexter (OD) and a superotemporal defect oculus sinister (OS). This visual field pattern strongly suggested a central lesion in the form of a junctional scotoma despite the noted absence of a RAPD on exam. Subsequent magnetic resonance imaging (MRI) revealed a large, right supraclinoid aneurysm, and the patient was treated accordingly. Through this case, one can appreciate how the classically described RAPD may not be present in all cases of CON if both optic nerves are affected. A provider must subsequently use other findings to distinguish between a central and peripheral nerve etiology. The importance of formal visual field testing in all cases of vision loss is highlighted to assess its etiology and aid in making this distinction between a central and peripheral lesion.

## 1. Introduction

Compressive optic neuropathy (CON) is a condition in which mechanical pressure placed upon the optic nerve results in damage. Given the optic nerve's anatomical course and neighboring structures, CON can produce a myriad of associated symptoms in addition to vision loss. In general, CON is a rare clinical condition, with about four in 100,000 cases per year [1]. Etiologies are vast, including tumor, aneurysm, thyroid disease, and inflammatory diseases, among others [1].

Overall, symptomatic CON by an internal carotid artery (ICA) aneurysm is rare. When symptomatic, the clinical course is chronic, with vision loss progressing over months to years [1]. Associated symptoms may include vision loss, headaches, photophobia, nausea, and vomiting [1]. Physical exam can reveal diplopia, dyschromatopsia, and a relative afferent pupillary defect (RAPD), in addition to visual field loss [1].

The optic disc may appear normal on examination early in the clinical course, so recognizing visual field defects, RAPD, and associated symptoms is important in diagnosing CON in a timely manner so that intervention can be most impactful [2, 3]. With asymmetric disease, the sensitivity of RAPD detecting an optic neuropathy has been cited to be as high as 98% [3]. However, this highly sensitive finding for unilateral optic nerve dysfunction is not a great indicator for bilateral disease. In this report, we focus on a case of bilateral CON caused by an ICA aneurysm. We discuss the importance of the visual field loss pattern and other significant clinical findings that raise suspicion for retrobulbar/chiasmal optic nerve disorders, such as CON and the importance of identifying these key findings.

## 2. Case Report

**Figure 1:**
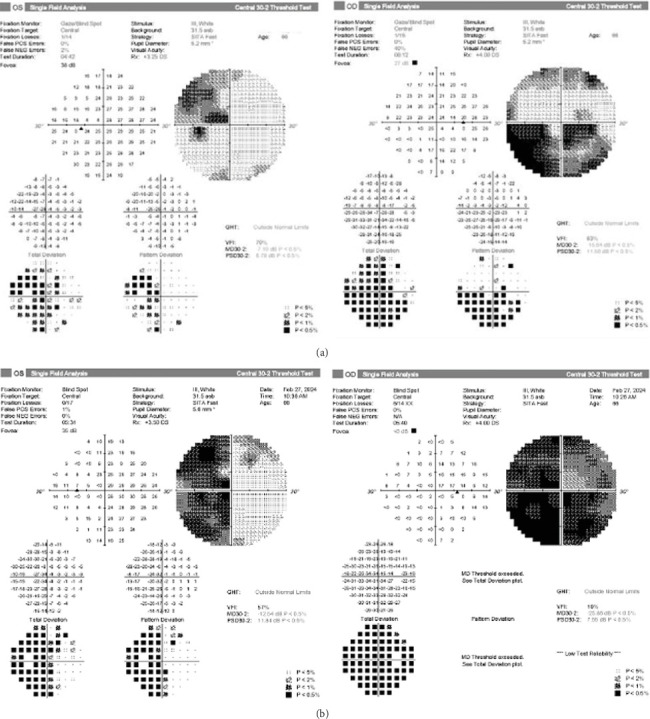
(a) Humphrey visual field 30-2 shows a superotemporal visual field defect OS and an inferior altitudinal defect OD, known as a junctional scotoma. (b) Humphrey visual field 30-2 shows a superior and inferior temporal defect respecting the vertical midline OS and 360 constriction OD.

**Figure 2:**
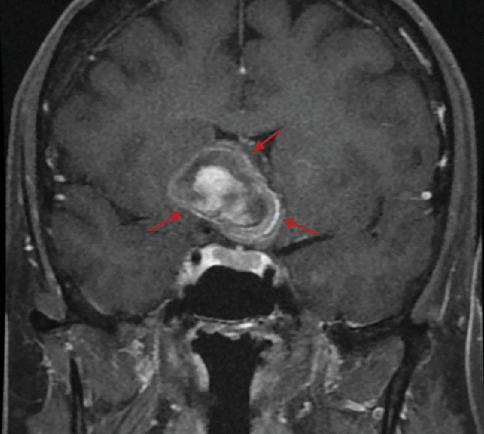
MRI, coronal T1 image showing a suprasellar mass with contrast enhancement consistent with active high flow into the lumen of a right supraclinoid carotid artery aneurysm. There is surrounding mass effect with compression of the right and left optic nerves and anterior chiasm with greater compression of the right optic nerve versus the left optic nerve.

A 66-year-old female with a history of chronic, long-standing primary open angle glaucoma in both eyes presented for routine follow-up. She had noticed headaches for a few months before her visit that had resolved without treatment and a decrease in vision that had started 3–4 months prior. On exam, her best corrected visual acuity had decreased from 20/30 to 20/200 oculus dexter (OD) and from 20/20 to 20/30 oculus sinister (OS) from her last interval appointment 6 months prior. Her intraocular pressure remained well controlled at 13 mmHg OD and 14 mmHg OS on timolol 0.5% twice daily oculus uterque (OU) and latanoprost nightly OU. Optical coherence tomography (OCT) retinal nerve fiber layer (RNFL) and ganglion cell layer (GCL) testing showed stable atrophy that was unchanged from her baseline testing due to her glaucoma. No RAPD was present; however, significant progression of visual field loss was noted on formal visual field testing with an inferior altitudinal defect OD and a superotemporal defect OS (Figure 1). Magnetic resonance imaging (MRI) (Figure 2) was obtained that showed a large right supraclinoid ICA aneurysm. The aneurysm exhibited significant mass effect, deformity of the floor and anterior wall of the third ventricle, and additional mass effect on the right and left optic nerve, with the right optic nerve being more severely affected. The patient underwent successful emergent coil embolization with pipeline flow diversion stenting of the aneurysm at an outside hospital. A follow-up visual field 2.5 weeks status postembolization showed 360 constriction OD and a superior and inferior temporal defect respecting the vertical midline OS (Figure 1).

## 3. Discussion

This patient's symptoms consisted of intermittent headaches, decreased visual acuity, and visual field deficits. The visual field defects offered the most important clue that her progressing vision loss was unlikely to be from her clinically well-controlled glaucoma. Although an altitudinal visual field defect is common in glaucoma, the striking respect for the vertical meridian evident on the pattern deviation in the left eye cannot be attributed to glaucoma—and when associated with an optic nerve–related visual field defect in the right eye, it should be recognized as a junctional scotoma [4]. A junctional scotoma occurs from anterior chiasmal lesions that affect the ipsilateral optic nerve and adjacent crossing fibers from the contralateral eye [2]. This visual field pattern directs the clinician to look for an anterior chiasmopathy—as was discovered with neuroimaging in this case.

As mentioned, the presence of a RAPD is a sensitive indicator of optic nerve dysfunction. When unilateral optic neuropathy suspicion is high, but no RAPD is revealed, bilateral disease was found in 65% of patients [3]. Since most CON is asymmetric, identifying a RAPD on exam is usually the clinical trigger for evaluation for possible CON or other retrobulbar optic neuropathies. However, in this rare case of a junctional scotoma with balanced optic nerve compression, no RAPD was found, demonstrating the importance of knowing the visual field status in addition to the RAPD. In our patient, although the aneurysm originated from the right supraclinoid segment of the carotid artery, the lesion was large enough to produce relatively equal visual field loss in both eyes, resulting in the absence of a RAPD.

Early identification of CON is critical for expeditious treatment initiation. Left untreated, CON can cause irreversible blindness, with acute cases potentially leading to such in as little as 2 h [5]. Delayed diagnosis and treatment could even lead to death from rupture of the aneurysm. Early decompression is preferred, as the visual outcome correlates with the time taken to decompress the nerve [1]. Management of CON can vary depending on the cause of compression [1]. As in our case, someone with a carotid aneurysm may undergo embolization of the artery. CON caused by recurrent tumors or trauma can be treated with radiation therapy or surgery, respectively [1]. Each treatment modality comes with risks but may be necessary to prevent further vision loss or even death.

## 4. Conclusion

This case illustrates a unique finding of bilateral CON secondary to a large, right supraclinoid ICA aneurysm. The typical RAPD exam finding associated with CON was absent due to the involvement of both optic nerves. The visual field defect revealed that the vision loss progression was unlikely to be attributed to glaucoma, but rather implicated a chiasmopathy prompting neuroimaging, demonstrating the importance of the visual field in evaluating all causes of vision loss.

## Data Availability

Data sharing is not applicable to this article as no new data were created or analyzed in this study.
